# Efficacy of intraperitoneal bupivacaine in laparoscopic bariatric surgery

**DOI:** 10.1016/j.amsu.2021.103229

**Published:** 2022-01-07

**Authors:** Tamer Saafan, Sabry Abounozha, Munzir Obaid, Mohamed Said Ghali

**Affiliations:** a General Surgery Department, Northumbria Healthcare NHS Foundation Trust, Northumbria, UK; b Acute Care Surgery Department, Hamad Medical Corporation, Doha, Qatar; c General Surgery Department, Ain Shams University, Cairo, Egypt

**Keywords:** Bupivacaine, Local anaesthesia, bupivacaine, Bariatric surgery, Bariatric procedure, Weight loss procedure

## Abstract

A best evidence topic has been constructed using a described protocol. The three-part question addressed was: In [patients undergoing bariatric surgery], is [intraperitoneal local bupivacaine during the operation ] associated with [ lower pain score and decrease in post operative pain medications]?

The search has been done and six randomized trial studies are considered to be appropriate to answer this question. The outcome assessed is the value of intraperitoneal bupivacaine in bariatric surgery in terms of effect on the pain score and post operative analgesia. We concluded that intraperitoneal bupivacaine causes improvement in both the pain score and post operative analgesia.

## Highlights

• Use of intraperitoneal bupivacaine, during bariatric procedures, improves post operative pain score.
• There is decrease in the post operative analgesia when intraperitoneal bupivacaine is used in the bariatric operations.
• Different ways can be used to apply intraperitoneal bupivacaine in the bariatric surgery field.

## 1 Introduction

This BET was constructed using a framework outlined by the International Journal of Surgery [1]. A BET provides evidence-based answers to the common clinical questions, using a systematic approach of reviewing the literature.

## 2 Clinical scenario

You are going to perform a laparoscopic bariatric surgery [eg. laparoscopic gastric bypass, laparoscopic sleeve gastrectomy, laparoscopic sleeve gastrectomy ] in an obese patient. You are anticipating that the patient will have post operative pain after such a procedure, and you understand that pain control is imperative for patient's recovery and length of stay [2]. This is especially important in the obese patients group, where improper pain control in a coexisting co-morbidities might increase the incidence of the post operative complications [2]. You discussed with a colleague whether or not to give an intraperitoneal local anaesthesia during the operation [bupivacaine], and you decided to conduct a systematic review to look for an evidence based answer for this technique.

## 3 Three-part question

In [patients undergoing bariatric surgery], is [intraperitoneal local bupivacaine during the operation ] associated with [lower pain score and decrease in post operative pain medications]?

## 4 Search strategy

The search was conducted as following: Embase 1974 to 06/December/2021 and MEDLINE® 1946 to 06/December/2021 using the OVID interface: [intraperitoneal] AND [local anaesthesia OR local anaesthetic OR bupivacaine] AND [bariatric surgery OR bariatric procedure OR weight loss procedure]. The search was limited to the English language and human studies. Studies which are non-randomized and conference abstracts were excluded from this review.

## 5 Search outcome

A total of 8020 papers were found using OVID. Out of those 8020, 8010 were excluded because they were irrelevant based on titles and abstracts or duplicates. Ten full-text articles were screened and examined for eligibility. Of those ten, six randomized trials papers were identified to provide the best evidence to answer the question.

## 6 Result

(Please refer to the Table 1).

## 7 Discussion

Obesity is a serious disease worldwide and bariatric procedures are becoming more popular as a tool to improve the quality of life and comorbidities [4,9]. Multiple studies has reported a decrease in the post operative pain and length of hospital stay with intraperitoneal local anaesthesia [4].

Safari et al., 2020 and Omer et al., 2019 [ 3,5] conducted a randomized controlled trial, to assess the efficacy of bupivacaine in the bariatric procedures, using 106 and 100 patients respectively. The two studies included different types of bariatric patients and included a mix of LSG, LRYGB and LMGB [Table 1]. Safari et al., 2020 installed 0.2% bupivacaine to wash the operated site before closure while Omar et al., 2019 installed 0.25% bupivacaine in the subdiaphragmatic space and patients held in Trendelenburg position for 5 min to enhance the effect of the intraperitoneal bupivacaine. Both [3,5] used paracetamol as part of the post op analgesia regime. Although Safari et al., 2020 used paracetamol infusion pump, Omar et al., 2019 used IV paracetamol and PCA morphine for the post operative pain control. Both studies showed favourable outcomes with improvement in the post operative pain score and reduction of post operative analgesia [3,5] for the intraperitoneal bupivacaine group when compared to the control group[patients who received intraperitoneal normal saline]. Safari et al. noted the maximum reduction in the pain score using intraperitoneal bupivacaine at the fourth hour postoperatively[pain score improved by 2.7 points]. Omar et al., 2019 showed similar findings with the maximum reduction in the pain score at the recovery followed by 4th hour post operatively, in which the pain score reduction was 1.12 and 0.86 respectively. Although safari et al. [3] noted that the improvement in the pain score lasted for 24 h, Omar et al. [5] noted that the effect of intraperitoneal bupivacaine on the pain score lasted for the first 6 h, with no difference in the pain score at 12 and 24 h. A smaller randomized trials from USA [ 6,7] [Table 1] showed also positive outcome with the use of intraperitoneal bupivacaine. Alkhamesi et al., 2008 [6] included only patients undergoing LRYGB while Sherwinter et al., 2008 [7] involved only patients having LAGB. Both Alkhamesi et al., 2008 and Sherwinter et al., 2008 reported statistically significant reduction in the pain score for the patients who received intraperitoneal bupivacaine, and the improvement in the pain score continued tell 24 h and 48 h respectively [6,7]. Moreover, in the Sherwinter et al. study [7], the improvement in the pain score in the intervention group was almost two fold that of the normal saline group, with the use of continuous infusion of intraperitoneal On-Q pump catheter containing 0.375% bupivacaine. Alkhamesi et al., 2008 [6] used a special device to aerosolize intraperitoneal 0.5% bupivacaine in the whole intraperitoneal cavity.

**Table 1:** Randomized trials examining the effect of intraperitoneal bupivacaine.

Author, date of publication, journal and country	Study type and level of evidence	Patient Type and number	Groups and Follow up	Type of IP local anaesthesia and mode of administration.	Port site skin wounds local anaesthesia infiltration for both groups	Primary outcomes	Secondary outcomes	Peri operative pain control/PONV control for both groups	Key results Primary outcome	Key results secondary outcome	Additional comments
Safari et al. [3] 2020 Iran	Randomized controlled trial	- Patients undergoing LSG, LRYGB and LMGB. - N:106 patients.	-IG: 54 patients, IPLA. -CG: 52 patients, IP normal saline.	- IG: 50 ml of 0.2% bupivacaine to wash operated site at end of surgery. - CG: 50 ml normal saline.	- Port wounds infiltrated with 2% lidocaine.	-Advantage of IPLA in post operative pain management in laparoscopic bariatric surgery.	NA	- 1 gm of paracetamol and 4 mg of ondansetron in the final 15 min of operation. - A continuous IV infusion pump containing 3 g of paracetamol was for 24 h. - Pethidine or morphine injections if VAS >3/patient's request.	- Pain score was statistically significant lower in IG compared to CG, at 1, 4, 8 and 24 h, 6.1 vs 7.4, 4.8 vs 7.5, 3.5 vs 5.7 and 2.5 vs 3.4, respectively. - Pethidine injection in the first 24 h after surgery was significantly less in IG compared to CG, 68.8 vs 103.9, respectively.	NA	- Single center - Pain assessed through visual analogue scale 0–10. - *Both pain score and post operative pain medications are less in the IG for 24 h.*
Schipper et al. [4] 2019 Netherlands	Randomized controlled trial.	- Patients undergoing LRYGB - N:127	- IG: 66 patients, IPLA. - CG: 61 patients, no IPLA.	-IG: 20 ml of 2.5% bupivacaine hydrochloride sprayed onto left side of the diaphragm. CG: no IPLA.	-Bupivacaine hydrochloride 20 ml of 2.5%	- Outcome of IP bupivacaine on postoperative pain after LRYGB.	- Post operative use of opioids. - Post operative use of antiemetics. - Length of stay	- Paracetamol 1 gm IV 2 h before surgery. - In the ward, all patients received 1000 mg paracetamol orally QID. - When pain score >4, 50 mg tramadol hydrochloride up to three times/day. Sufentanil injection may be added as needed.	- No statistically significant reduction of postoperative abdominal or shoulder pain in IG compared to CG.	- No statistically significant difference in terms of decrease of post operative opioids or antiemetics or length of stay.	- Single center. - Age is statistically significant higher in IG compared to CG, mean 46.2 vs 42.3 years. - Pain assessed through VAS. - *No improvement in pain score or post operative pain medications.*
Omar et al. [5] 2019 Bahrain	Randomized controlled trial	- Patients undergoing LSG, LSG + C, LRYGB and LMGB - N:100 patients	- IG:50 patients, IPLA. - CG: 50 patients, IP normal saline	-IG: 40 ml bupivacaine 0.25%) given in the subdiaphragmatic space and patients were held in Trendelenburg's position for 5 min. - CG: 40 ml normal saline.	- Bupivacaine 20 ml 0.25%.	- Efficiency of IP bupivacaine after bariatric procedures for pain management.	- PONV - Shoulder tip pain	- Paracetamol 1 gm IV Q 6 h + PCA morphine.	- Pain scores statistically significant lower in intervention group compared to control at recovery, 2, 4 and 6 h after surgery, with mean 2.78 vs 3.9, 1.84 vs 2.38, 1.68 vs 2.54 and 1.6 vs 2.14, respectively. No significant difference at 12 and 24 h post surgery. - Rescue analgesia is significantly less in intervention compared to control group, 16% vs 54% respectively. - Lower morphine consumption via PCA in intervention compared to control group, 13.24 vs 16.9, respectively.	- No statistically significant difference for PONV and shoulder tip pain in both groups. –	- Single center - Pain assessed through VAS. - *Pain score has improved in IG group for first 6 h post operative.* - *Post operative analgesia is less also in the IG group.*
Alkhamesi et al. [6] 2008 USA	Randomized controlled study	- Patients undergoing LRYGB - N:50	- IG: 25 patients, IPLA - CG: 25 patients, IP normal saline.	- IG: 10 mL, (50 mg) of IP aerosolized 0.5% bupivacaine through a special device to ensure that intraperitoneal space is completely covered. - CG: 10 mL of aerosolized IP normal Saline.	−30 mL of 0.5% bupivacaine with epinephrine (150 mg of bupivacaine)	-Examine safe use of the aerosolization technique in bariatric surgery and to assess its efficacy in post operative pain management.	NA	-PCA containing dilaudid or morphine	Pain score was statistically significant less in the IG compared to CG, p = 0.01. Effect on pain score continued tell 24 h.	NA	- Single center. - Small sample size. - Pain assessed through simple pain score by a nurse ranging from 0 to 10 similar to VAS. - *Pain score is better in IG group.* - No statistically significant difference in post operative analgesia
Sherwinter et al. [7] 2008 USA	Randomized controlled study	- Patients undergoing LAGB. - N:30 patients.	- IG: 14 patients, IPLA. - CG: 15 patients, IP normal saline.	- IG: continuous infusion of an intraperitoneal On-Q pump catheter containing 0.375% bupivacaine at 7.5 mg/h for a daily dosage of 180 mg with a total of 360 mg over 48 h. Catheter placed at site of maximum dissection. - CG: intraperitoneal On-Q pump containing 0.9% normal saline.	− 20 ml of 0.5% bupivacaine	- Efficacy of IPLA through on-Q system in terms of pain score (VAS).	- Shoulder tip pain. - PONV and need for antiemetics. -Post operative morphine requirements.	- Postoperative analgesia for all patients was given as IV ketorolac QID and IV morphine as needed for pain. - On-Q pumps were removed at 48 h postoperatively.	- At 6 h, VAS was statistically significant lower in the IG compared to CG, 1.8 vs 3.5, respectively, and remained significantly lower tell end of study at 48 h, 0.93 vs 3.0, respectively.	- No statistically significant difference between two groups in terms of shoulder pain, PONV and post operative morphine requirements.	- Single center. - Small sample size. - Pain assessed through VAS. - All measurements of different primary and secondary outcomes done at 30min and 6,12,24 and 48 h. - *Pain score is better in IG group.* - *No difference in post operative analgesia.*
Symons et al. [8] 2007 USA	Randomized controlled study.	- Patients undergoing LRYGB. - N:133 patients.	- IG: 65 patients, IPLA. - CG: 68 patients, IP normal saline.	- IG: 15 ml of IP 0.5% bupivacaine. After pneumoperitoneum, it was sprayed through an instrument aimed at the oesophageal hiatus - CG: 15 ml IP normal saline, using same technique as IPLA.	− 0.5% bupivacaine with epinephrine.	To assess: - Post operative narcotic use - VAS pain score. - Incentive spirometry volumes. - Antiemetic use	NA	- In the post operative period all patients had standardized medications, including hydromorphone PCA and IV metoclopramide as needed for nausea. - On the first postoperative day, patients were offered oral hydrocodone/paracetamol as needed for pain, 12–20 h post operative.	- IG showed statistically significant difference in postoperative hydrocodone/paracetamol compared to CG, 23.8 vs 33.7 ml, respectively. - No statistically significant difference in terms of length of stay, pain score and incentive spirometry	NA	-Single center. - IPLA given at beginning of the operation not at the end as others. -Pain assessed through VAS. *-no difference between IG and CG in terms of pain score.* *-IG group had less post operative analgesia requirements*.

LSG: laparoscopic sleeve gastrectomy, LSG + C: laparoscopic sleeve gastrectomy with cardioplasty, LRYGB: Laparoscopic roux en Y gastric bypass, LMGB: laparoscopic mini-gastric bypass, LAGB: laparoscopic adjustable gastric banding, N: number, IP: intraperitoneal, IPLA: intraperitoneal local anaesthesia, VAS: Visual Analogue Scale measuring pain intensity 0–10 with 0 considered as no pain and 10 considered as worst pain imaginable, NA: not applicable/not mentioned, PCA: patient controlled analgesia, PONV: post operative nausea and vomiting, IG: intervention group, CG: control group.

On the other hand, Symons et al., 2007 [8] and Schipper et al., 2019 [4] reported no difference in the pain score with the application of intraperitoneal bupivacaine compared to intraperitoneal normal saline [Table 1]. Both studies included patients undergoing only LRYGB and both used a mix of paracetamol and opiates post operatively. Sample size in Symons et al., 2007 and Schipper et al., 2019 were similar, 133 and 127 respectively [4,8]. In Symons et al., 2007 [8], 0.5% intraperitoneal bupivacaine was sprayed at the oesophageal hiatus, while Schipper et al., 2019 [4] sprayed 2.5% bupivacaine at the left side of the diaphragm hiatus. Although there was no reduction in the pain score in both studies, Symons et al., 2007 [8] showed that patients who received intraperitoneal bupivacaine had less post operative analgesia requirements compared to the normal saline group.

In summary, four randomized trials showed improvement in the pain score in the bariatric operations [3,[5], [6], [7]], and two of which [3,5] showed additional reduction in the post operative analgesic requirement of the patients. While the other two studies [4,8] didn't show statistically significant reduction in the pain score with the use of intraperitoneal bupivacaine, Symons et al., 2007 [8] reported statistically significant reduction in the post operative analgesia demands in the patients who received intraperitoneal bupivacaine. From the above findings, it is evident that intraperitoneal bupivacaine does help with the pain management in bariatric patients by either reducing the pain score or reducing the post operative analgesics.

### 7.1 Limitations of this review

1. There are different amounts and concentrations of bupivacaine used in the intervention groups in the above six randomized trials.
2. Intraperitoneal bupivacaine was applied using different techniques among the randomized trials in this review.
3. There is heterogenicity among the type of bariatric procedures included in each study, as some studies included one specific type of patients undergoing bariatric procedures [Table 1] and others included a mix of patients undergoing different bariatric procedures [Table 1].
4. There are different post operative analgesia medications regime among the different randomized trials.

### 7.2 Clinical bottom line

There is a good evidence that intraperitoneal bupivacaine causes improvement in the control of postoperative pain after bariatric procedures, in terms of reduction of the pain score and post operative analgesia.

## Ethical approval

Not applicable.

## Sources of funding

None.

## Author contributions

TS: devised the idea of the study, conducted literature search and wrote the paper.

SA: assisted in literature search and collecting the data.

MO: assisted in literature search and writing the paper.

MSG: assisted in literature search editing and writing the paper.

## Guarantor

Tamer Saafan, Sabry Abounozha, Munzir Obaid, Mohamed Said Ghali

## Trial registry number

1. Name of the registry: Not applicable
2. Unique Identifying number or registration ID: Not applicable
3. Hyperlink to your specific registration (must be publicly accessible and will be checked): Not applicable

## Consent

Not applicable.

## Declaration of competing interest

None.
